# hnRNPA2B1 Promotes Colon Cancer Progression via the MAPK Pathway

**DOI:** 10.3389/fgene.2021.666451

**Published:** 2021-09-22

**Authors:** Jingzhi Tang, Zhimin Chen, Qi Wang, Weijie Hao, Wei-Qiang Gao, Huiming Xu

**Affiliations:** ^1^State Key Laboratory of Oncogenes and Related Genes, Renji-MedX Clinical Stem Cell Research Center, Ren Ji Hospital, School of Medicine, Shanghai Jiao Tong University, Shanghai, China; ^2^Colorectal Surgery Department, Changhai Hospital, Naval Medical University, Shanghai, China

**Keywords:** colon cancer, hnRNPA2B1, cell proliferation, alternative splicing, N6-methyladenosine

## Abstract

HNRNPA2B1, an RNA-binding protein, plays a key role in primary microRNA processing, alternative splicing, mRNA metabolism and transport. Interestingly, hnRNPA2B1 also works as an N6-methyladenosine (m6A) reader and is critical during tumorigenesis of various tissue types. However, its role in colon cancer is still unclear. In this study, we aimed to elucidate the biological functions of hnRNPA2B1 and to explore its underlying mechanisms in colon cancer. We examined the expression of hnRNPA2B1 in Oncomine and TCGA databases. Then verified the findings in colon cancer cells and clinical samples with western blotting and immunohistochemistry (IHC). We used CRISPR/Cas9 directed gene editing to knockout hnRNPA2B1 expression in human colon cancer cell line SW480 and HCT-116 and carried out both *in vivo* and *in vitro* experiments. The results were further confirmed by RNA-seq analyses. We found that hnRNPA2B1 significantly promoted colon cancer cell proliferation both *in vitro* and *in vivo*, while knockout of hnRNPA2B1 induced apoptosis and cell cycle arrest in SW480. RNA-seq analyses revealed that the ERK/MAPK pathway was activated by hnRNPA2B1 upregulation. In addition, both hnRNPA2B1 and MAPK pathway were activated in clinical colon cancer specimens and positively correlated. Mechanistically, hnRNPA2B1 appeared to be an upstream regulator of the ERK/MAPK pathway and inhibition of MAPK signaling blocked the effects of hnRNPA2B1. Taken together, our data demonstrated that the RNA-binding protein hnRNPA2B1 promotes cell proliferation and regulates cell cycle and apoptosis of human colon cancer by activating the ERK/MAPK signaling, which may provide a new insight into the development of hnRNPA2B1 as a potential therapeutic target for treatment of colon cancer.

## Introduction

Colon cancer is the fourth most common malignancy and has the second-highest mortality rate with more than 53,200 estimated deaths occurring in the United States during 2020 (Siegel et al., 2020). Nowadays, the increasing incidence of colon cancer is more likely to accelerate rapidly due to changes in lifestyle, nutritional habits, population aging, and other factors (Chen et al., 2018). In the patients with colon and rectal cancer together (Colorectal cancer, CRC), despite significant differences in anatomy and gene expression, more than 50% have advanced disease (stage III or IV) at the time of diagnosis and 5-year survival rate among these patients is no greater than 40%, suggesting that early diagnosis is crucial for patient survival (Chen et al., 2016). In addition, about 50% of CRC patients develop organ-specific metastasis in the liver during the course of disease, which is the main cause of death in patients with CRC (Kopetz et al., 2009). At present, surgery remains the principal treatment of colon cancer. Since the quality of life in patients with cancer is often greatly affected by the surgery, it is necessary to understand how colon cancer is developed so to come up with novel therapeutic strategies for the diagnosis and treatment of the disease.

There are more than 100 modifications in RNA, which mainly occur in the nucleus. M6A modification is the most common one, accounting for more than 50% (Zhang et al., 2019; Zhang H. et al., 2020). It can regulate the stability, positioning, transport, splicing, and translation of RNA at the post-transcriptional level (Wang et al., 2014; Adhikari et al., 2016; Visvanathan et al., 2018). It also plays an important role in tumorigenesis and progression (Zhang B. et al., 2020). To date, 21 genes involved in the regulation of m6A modification have been discovered, which act as either oncogenes or tumor suppressor genes in malignant tumors (He et al., 2018; Weng et al., 2018), and mainly include three types of molecules: methyltransferases (Writers), demethylases (Erasers), and m6A binding proteins (Readers). As a member of readers, hnRNPA2B1 is an important RNA-binding protein, responsible for “reading” the information of RNA methylation modification, and participating in downstream RNA translation, degradation (Alarcon et al., 2015).

As a member of hnRNP family (Geuens et al., 2016), hnRNPA2B1 contributes to telomere function, mRNA translation, splicing, correct localization of transcripts, loading of exosomes and etc. (Streitner et al., 2012). More importantly, hnRNPA2B1 plays a significant role in many different types of tumors and it can act as an oncogene in the development of certain tumors (Wang et al., 2018). For example, in bladder cancer (BCa), hnRNPA2B1 can mediate LNMAT2 packaging into exosomes, ultimately resulting in lymphangiogenesis and lymphatic metastasis (Chen et al., 2020). Yang et al. reported hnRNPA2B1 promotes ovarian cancer cell proliferation and progression by enhancing Lin28B expression (Yang et al., 2020). In lung cancer cells, hnRNPA2B1 modulates EMT through the regulation of E-cadherin expression (Tauler et al., 2010). However, its role in colon cancer is still unclear.

The ERK/MAPK signaling, which is a signal transduction pathway that regulates a vast array of physiological functions (Liu et al., 2018), including cell proliferation, differentiation, apoptosis and a component of the RAS/RAF/MEK/ERK MAP kinase (MAPK) pathway. The activation of ERK/MAPK signaling plays a vital part in progression of colon cancer (Fang and Richardson, 2005; Tang et al., 2019). However, whether the underlying mechanism involved in the activation of ERK/MAPK pathway in colon cancer is not well-determined.

In the present study, we compared expression of hnRNPA2B1 in colon cancer and matched adjacent normal tissues from databases and clinical samples and found that hnRNPA2B1 was upregulated in colon cancer, which markedly promoted colon cancer cell proliferation *in vitro* and *in vivo.* By performing RNA-seq analyses, we observed that activation of ERK/MAPK pathway was strongly associated with expression of hnRNPA2B1. In addition, knocked out experiments revealed that hnRNPA2B1 positively regulated the ERK/MAPK pathway. These data are helpful for our understanding of colon cancer development, diagnosis and therapeutic intervention of colon cancer.

## Materials and Methods

### Gene-Expression Data Processing

Gene expression data were obtained from the colon cancer cohorts from the Oncomine and TCGA database. The TCGA database included 44 normal colon samples and 568 colon cancer samples. The TCGA data were downloaded from TCGA-colon cancer dataset^1^ and analyzed with the statistical programming R language.

### Immunohistochemistry

Tumor tissues were fixed with 4% PFA and embedded in paraffin. The sections of 3–4 microns thick were baked on a hot plate at 60° for 1 h, followed by dewaxing (5 min for 3 times) in xylene, and dehydration in 100, 90, 80, and 70% ethanol, respectively for 5 min. Antigen retrieval was conducted in microwave for 20 min in citrate buffer (pH 6.0) and endogenous peroxidase was blocked by 3% hydrogen peroxide in methanol for 10 min. Then, sections were permeabilized in 0.3–0.5% Triton X-100 for 10 min, blocked with 5% normal donkey serum (Jackson# 017–000–121) in PBST for 1 h, and incubated in primary antibody (1:400) overnight at 4°C. Sections were washed in 1 × PBST (PBS containing 0.05% Tween-20) the next day for 10 min, incubated with the appropriate secondary antibody for 1 h at room temperature and washed again. The staining was developed with DAB (gene tech# GK347011) and counterstained with hematoxylin. Next, the sections were dehydrated with sequential concentration of ethanol (the ethanol concentration was raised from 70, 80, 90, and 100%), cleared in xylene and finally sealed with neutral resin at the end.

### Western Blotting

The specimens were washed repeatedly with cold PBS (Thermo Fisher Scientific #21,600–069) twice and then immersed in RIPA Lysis buffer (Thermo Fisher Scientific #89,901) which was diluted with a protein inhibitor cocktail 1:100 in 1× RIPA Lysis buffer, cleaved for 15 min then collected the supernatant. The protein concentration was determined by BCA assay (Thermo Fisher Scientific #23,227). After that, the supernatant was mixed with 6X SDS loading buffer, and boiled for 10 min, separate by SDS–PAGE. Lysates were then run on an SDS/10–15% polyacrylamide gel (Bio-Rad) and transferred onto 0.45μm PVDF membranes (Invitrogen), blocked with by 1 × TBST buffer containing 5% skim milk at room temperature for 1 h. The membrane was incubated with the first antibody (1:1,000 dilution) at 4°C overnight, rinsed with TBST, and then incubated with the second antibody (1:8,000 dilution) for 1 h at room temperature, washed by TBST buffer solution at 3 times every 5 min, detected by the ECL reagent (Millipore#WBKLS0500), and directly imaged and digitalized using a BioRad VersaDoc 4,000 imaging system and quantified by the Quantity One software (BioRad, CA, United States).

### Establishment of Stable hnRNPA2B1 Knockout Cell Lines

We constructed Lenti-CRISPR-V2 (Addgene#52,961) control empty vector and sgRNA directed against hnRNPA2B1. The following primers were used to examine sgRNA plasmid: sgRNA-F1: CACCGACTCTCCCATCAATTGAATG, sgRNA-R1: aaacCATTCAATTGATGGGAGAGTC; sgRNA-F2: CACCGGGAAAGCTTACAGACTGTG, sgRNA-R2: aaacCACAGTCTGTAAGCTTTCCC. We constructed two overexpression plasmids, including hnRNPA2B1 (OE-1) and hnRNPA2 (OE-2). Codon optimization was employed in constructing the hnRNPA2B1 plasmid. The hnRNPA2 overexpression plasmid was constructed by amplifying the hnRNPA2 fragment through gene synthesis (Gupta et al., 2020). Lentiviral plasmids were transfected into HEK-293T cells along with the packaging plasmid psPAX2 (Addgene#12,260) and envelope plasmid pMD2.G (Addgene#12,259) using the Lipofectamine 3,000 reagent (Invitrogen, Carlsbad, CA, United States). The virus supernatant was collected after 48 or 72 h and then mixed with 8 μg/ml polybrene and directly added to cells for infection. After 48 h of infection 2 μg/ml puromycin was added for cell selection until the establishment of stable knockout cell lines. Finally, the efficiency of stable cell lines was analyzed by western blotting.

### Cell Culture

Human colon cancer cell lines SW620, SW480, HCT-8 and HCT-116 were from the Cell Bank of Chinese Academy of Sciences (Shanghai, China). Cell culture medium was RPMI 1,640 medium (gibco#C22400500BT) supplemented with 10% FBS (gibco#10099-141) and 1% Penicillin/Streptomycin (gibco#15140) and cell cultures were maintained with 5% CO_2_ at 37°C (Thermo Fisher Scientific). Cells were passaged or expanded at 90% confluency, washed with PBS twice, and then digested and centrifuged for 5 min at 1,000 rpm. The cells then re-suspended and seeded in culture dishes, with media change performed every 2 days and cryopreserved in a mixture of 90% FBS and 10% DMSO (Gibco#D2650).

### Cell Cycle and Apoptosis Analyses

Cell cycle distribution and apoptosis after transfection were detected by flow cytometry, which was performed on BD Biosciences LSR Fortessa. For cell cycle analyses, cells with 90% density were harvested and fixed with 75% ethanol for 2 h at −20°C. Then cells were taken for PI staining and cell cycle was analyzed using flow cytometry. Cell apoptosis was analyzed by flow cytometry according to manufacturer’s protocols with Annexin V-APC and 7-AAD staining (Biolegend#640930). Channel FL2-A/PE for cell cycle detection and APC/PerCP for apoptosis detection. Flowjo software was used for data analyses.

### Colony Formation Assay, CCK-8 Assay and Sphere-Forming

To examine colony formation ability, transfected cells were seeded in six well-plate for 12 days (1,000 cells were seeded into each well of a six-well plate). Then, the cells were fixed with 4% paraformaldehyde (PFA), stained with 0.2% crystal violet (Beyotime, Shanghai, China) and photographed. In the meantime, cell proliferation was further detected by the CCK8 kit at 24, 48, and 72 h. Experiments were repeated three times independently. For Sphere-forming assays, 500 cells were cultured in 6-well Ultra-low Attachment surface plate (Corning#3,471) with serum-free DMEM medium containing for 2 weeks. Cells were fed with 1 ml of medium every 2 days. Photos were taken using an inverted microscope and the sphere numbers in each well were quantified. GraphPad Prism version 8.0 was used for statistical analyses.

### *In vivo* Tumor Growth and Metastasis

Four-week-old male BALB/c nude mice (purchased from Lingchang company) were randomly divided into three groups, each group has five mice. Each of the mice was injected subcutaneously on the right lateral back with 1 × 10^6 of each lentivirus infected SW480 cells in which hnRNPA2B1 was knocked out or negative control cells. Mice were killed at day 28, and tumors were then isolated, photographed. All protocols for animal use and euthanasia were reviewed and approved by RenJi Hospital Institutional Animal Care and Ethics Committee (RJ2020-0601).

### RNA-Seq

RNA sequencing was performed using the Illumina Hiseq-PE150 platform and analyzed with the statistical analyses in this study were generated by R- 4.0.3. Differentially expressed genes (DEGs) were identified using edgeR package. Genes with log-fold change > 1.8 and false discovery rate (FDR) < 0.05 were considered differentially expressed. Gene ontology (GO) analyses, Kyoto Encyclopedia of Genes and Genomes (KEGG) pathway enrichment analyses and Gene Set Enrichment Analyses (GSEA) were performed using R package clusterProfiler to observe the functions of the DEGs. We used the STRING database to predict protein-protein interactions among the up-regulated genes, then further processed the data with the Cytoscape software (version 3.7.2).

## Results

### hnRNPA2B1 Expression Is Selectively High in Colon Cancer

**FIGURE 1:**
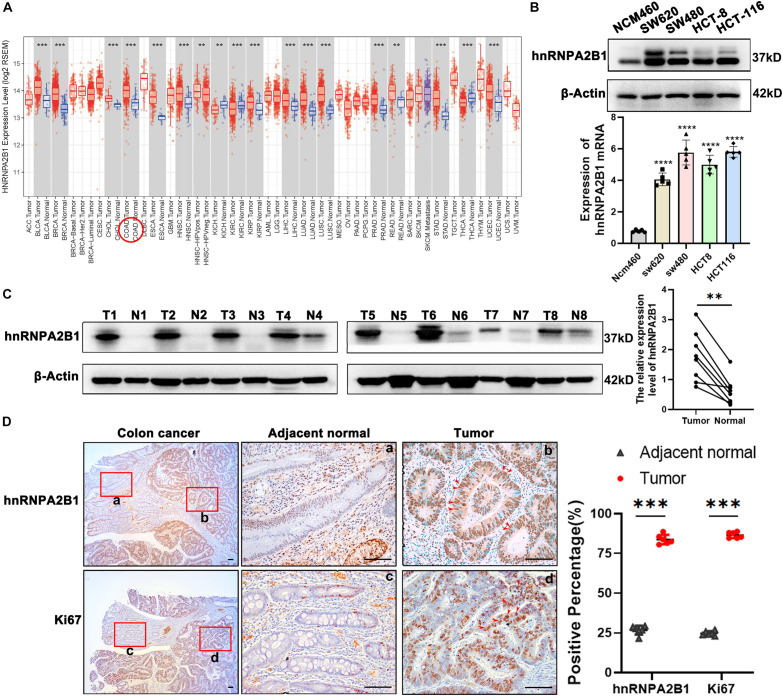
hnRNPA2B1 is highly expressed in colon cancer. **(A)** Gene expression profiles in TCGA database. COAD, Colorectal adenocarcinoma; READ, Rectum adenocarcinoma. **(B)** Protein and mRNA level of hnRNPA2B1 in normal intestinal epithelial cells NCM460 and four colon cancer cell lines including SW620, SW480, HCT-8, and HCT-116. The expression level of hnRNPA2B1 in SW480 and HCT-116 is higher than the other cell lines, so we chose SW480 and HCT-116 for further study. **(C)** Western blotting analyses of hnRNPA2B1 in colon cancer tumor tissues (T) and normal tissues (N) (*n* = 8). Semi-quantitative analyses of protein were carried out with the Image Lab software and paired *t*-test was employed for the statistics. **(D)** Immunohistochemistry and semi-quantitative analyses of hnRNPA2B1 and Ki67 expression in colon cancer (scale bar: 100μm). Statistical significance was assessed using 2-tailed Student’s *t*-test. **p < 0.01 and ***p < 0.001.

To investigate whether hnRNPA2B1 plays a role in tumorigenesis, we checked varied databases to identify expression level of hnRNPA2B1 in human tumors. We found that hnRNPA2B1 was highly expressed in various cancers in Oncomine and TCGA databases (Supplementary Figure 1A and Figure 1A), which was consistent with what was previously reported (Liu et al., 2020; Yang et al., 2020). We further investigated the prognosis of hnRNPA2B1 and found that patients with higher hnRNPA2B1 expression showed a worsen survival (Supplementary Figure 1B).

As hnRNPA2B1 is one of m6A-related genes, we then turned our attention to expression of all 21 m6A-related genes in colon cancer. We downloaded and compared them from The Cancer Genome Atlas (TCGA) and found that most of m6A-related genes were expressed at much higher levels in colon cancer, compared to normal tissues, and that of the 21 m6A-related genes. These data suggest an uniqueness of the selective high expression of hnRNPA2B1 in colon cancer (Supplementary Figure 1C).

### hnRNPA2B1 Is Upregulated in Colon Cancer Compared With Adjacent Normal Tissues.

To validate the selective high expression levels of hnRNPA2B1, we performed Western blot or RT-qPCR analyses in 4 colon cancer cell lines including SW620, SW480, HCT-8, HCT-116 and normal intestinal epithelial cells NCM460 as well as clinical colon samples. As shown in Figure 1B, consistent with the above described data-mining, the expression levels of hnRNPA2B1 were significantly elevated in all 4 colon cancer cell lines examined compared to normal intestinal epithelial cells NCM460. In addition, we examined and compared the expression of hnRNPA2B1 in 8 pairs of colon cancer tissues and normal colon tissues. Data from analyses of western blot indicated that hnRNPA2B1 expression level was significantly upregulated in human colon carcinoma compared with normal colon tissues (Figure 1C). Similarly, higher expression levels of hnRNPA2B1 were also observed in tumor tissues (Ki67 positive) of colon cancer patients by IHC assay (Figure 1D). Thus, hnRNPA2B1 was upregulated in established colon cancer lines and colon cancer tissues.

### hnRNPA2B1-Knocked Out Inhibits Cell Proliferation *in vitro* and *in vivo*

**FIGURE 2:**
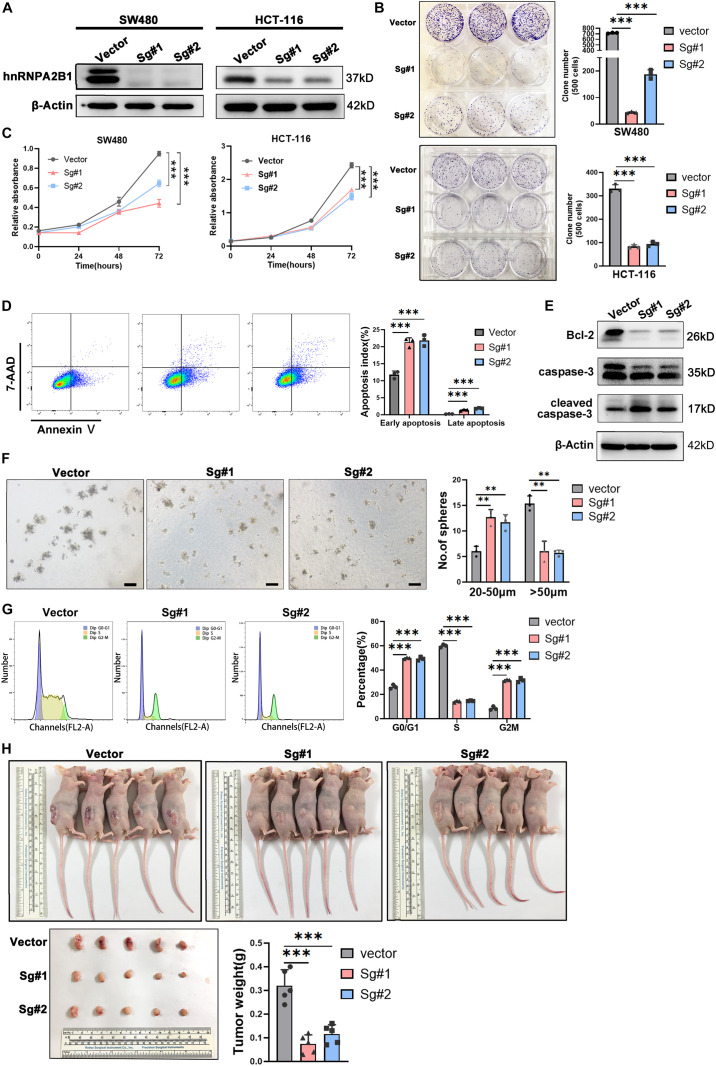
hnRNPA2B1 knockout inhibited colon cancer cell proliferation and cell cycle progression *in vitro* and *in vivo*. **(A)** Stable knockout of hnRNPA2B1 in SW480 and HCT-116 cell lines by two different SgRNA sequences (Sg#1 and #2). **(B)** Colony formation assay showed that colony formation ability in SW480 and HCT-116 hnRNPA2B1 stable knockout cell lines was decreased compared with the control group (^∗∗∗^*P* < 0.001). **(C)** CCK-8 assay demonstrated that the proliferation rate in SW480 and HCT-116 hnRNPA2B1 stable knockout cell lines were significantly decreased compared with the control group (^∗∗^*P* < 0.01; ^∗∗∗^*P* < 0.001). **(D)** Cell apoptosis was measured by Flow Cytometry. The cells stained with annexin V/7-Aminoactinomycin D (7-AAD). **(E)** The expression of apoptosis-related proteins was detected by Western blot analyses. **(F)** Sphere-forming capability was evaluated by plating cells in low adhesion plastic (scale bar: 100 μm). **(G)** The cell cycle pattern (G0/G1, S and G2/M) was also determined by flow cytometry. Three individual experiments were performed. The cell cycle was assessed using PI staining. **(H)** Tumorigenesis assay was performed by subcutaneous injection of hnRNPA2B1 stable knockout SW480 cells into three flanks of BALB/c nude mice. The nude mice were harvested and tumors were removed and photographed at 28th day after injection. All the statistical significances were assessed using two-way ANOVA.

To determine possible functions of hnRNPA2B1 in colon cancer, we used CRISPR/Cas9 directed gene editing to knockout hnRNPA2B1 expression in human colon cancer cell lines SW480 and HCT-116. Western blot analyses confirmed that hnRNPA2B1 expression was essentially abolished in colon cancer cell line in which hnRNPA2B1 was knockout all (Figure 2A). At first, we assessed proliferation ability of hnRNPA2B1 knockout group and control group by performing clone formation assay and CCK8 assay (Figures 2B,C). Proliferation of hnRNPA2B1 knockout cells was significantly lower than the control group, indicating that the knockout of hnRNPA2B1 can impair the proliferation ability of colon cancer cells *in vitro*. Then the cells were trypsinized and equal cell numbers were used to process for apoptosis assay by flow cytometry. The results showed that the apoptosis rate of colon cancer cells in the KO group was significantly increased (Figure 2D). Consistently, results of western blotting assays showed that expression of apoptotic protein cleaved caspase-3 was increased, and the decreased Bcl-2 level confirmed this phenomenon, which shows increased apoptosis in KO cell lines (Figure 2E). To further confirm these results, we also performed sphere-forming assay, which showed that knocking out hnRNPA2B1 impaired the ability of these SW480 cells to form tumor spheres (Figure 2F). In addition, when cell cycle was analyzed by flow cytometry (Figure 2G), the knockout cells were arrested at G0/G1 phase. Collectively, these experiments indicated that knockout of hnRNPA2B1 in colon cancer cells decreases cell proliferation and increases apoptosis *in vitro*.

Further investigation needs to be conducted to confirm the oncogenic role of hnRNPA2B1 in colon cancer, so subcutaneous tumor formation experiments were performed in nude mice. Mice were euthanized at the 28th day and the tumors were removed, photographed and weighed (Figure 2H). The results showed that tumors generated by hnRNPA2B1 knockout cells were significantly smaller than control, indicating that hnRNPA2B1 facilitated colon cancer tumorigenesis *in vivo.*

### hnRNPA2B1 Promotes the Development of Colon Cancer by Regulating the ERK/MAPK Pathway

**FIGURE 3:**
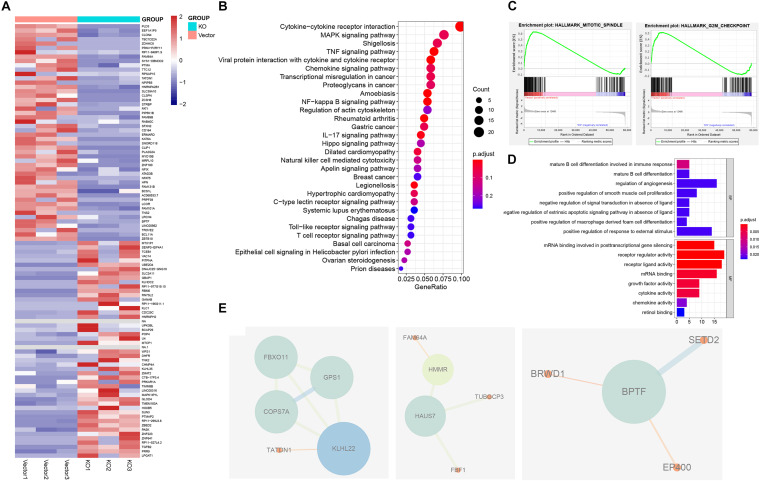
Identification of hnRNPA2B1 targets via RNA-seq. **(A)** Heatmap showing differential gene expression profiles in KO cell lines relative to the vector group. **(B)** KEGG pathway analyses of differentially expressed genes between hnRNPA2B1 knockout and control SW480 cells. **(C)** GSEA analyses in hnRNPA2B1 knockout colon cancer cells versus control cells. **(D)** Top 8 down-regulated genes items of GO analyses. The *x*-axis presents enrichment extent of the target genes in a specific category. BP, biological process; CC, cellular component; MF: molecular function. DEG, the differentially expressed gene; GO, Gene Ontology. **(E)** Three PPI modules were presented. A larger size of nodes represents a higher degree. PPI, protein-protein interaction.

To investigate possible molecular mechanisms of hnRNPA2B1 involved in colon cancer pathogenesis and progression, we performed the RNA-seq analyses. Then the gene expression data were analyzed using the R package. Heatmap of the differential expression of genes in colon cancer between control and KO group, revealed significant differences in the gene expression profile between the two groups. We utilized the Bioconductor package edgeR for differential analyses. In heatmap (Figure 3A), differentially expressed genes (DEGs) were determined (corrected *P* < 0.05, logFC > 1.8 or logFC <–1.8). Genes with higher expression are shown in red, lower expression in blue. The Kyoto Encyclopedia of Genes and Genomes (KEGG) pathway and Gene Ontology (GO) enrichment analyses were used to determine the significant gene function based on the DEGs obtained from RNA-seq experiments. KEGG revealed that these genes were involved in a variety of signaling pathways, including the cytokine-cytokine receptor interaction, the TNF signaling pathway, and the NF-kappa B signaling pathway, while the most noticeable difference genes change involved tumor was the MAPK pathway (Figure 3B). GO analyses showed that many genes involved in biological processes such as alternative splicing was upregulated in high hnRNPA2B1 expression of control group as compared to the hnRNPA2B1 knockout group (Figure 3D). Meanwhile, GSEA analyses indicated control group had an obvious enrichment in hallmark mitotic spindle and G2M checkpoint (Figure 3C). Protein-protein interaction network (PPI) revealed that functional enrichment of alternative splicing increased in the control group. PPI modules demonstrated that BPTF, KLHL22, and HAUS7 were the three alternative splicing hub genes among this dataset (Figure 3E). Interestingly, we have also observed the effect of hnRNPA2B1 on alternative splicing in colon cancer from TCGA (Supplementary Figure 2A). This is consistent with what we have described above.

**FIGURE 4:**
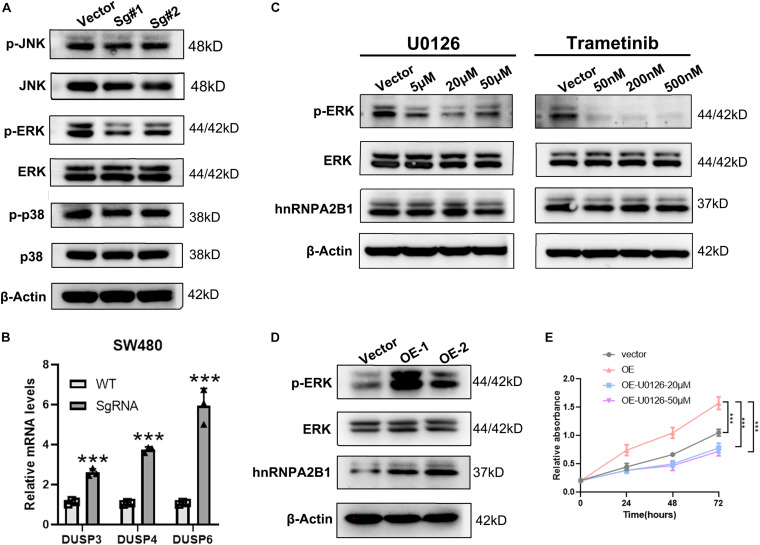
hnRNPA2B1 promotes the development of colon cancer by regulating the ERK/MAPK signaling pathway. **(A)** Western blot analyses was applied to detect the expression of MAPK pathway related proteins. **(B)** RT-qPCR analyses of expression of DUSP3/DUSP4/DUSP6. **(C)** The MEK inhibitors U0126 and Trametinib induced an obvious decrease in the expression of phospho-ERK. **(D)** Overexpression of hnRNPA2B1 in HCT-8 cells. OE-1, overexpression of hnRNPA2B1; OE-2, overexpression of hnRNPA2. **(E)** CCK-8 assay demonstrated the proliferation rate in HCT-8 cell lines. Vector, HCT-8WT; OE, hnRNPA2B1-overexpression; U0126, MAPK inhibitor (**P < 0.01; ***P < 0.001). Data were analyzed by two-way ANOVA.

Given that, the above experiment showed that the MAPK signaling pathway was activated when hnRNPA2B1 is expressed. To determine whether MAPK pathway is downstream of hnRNPA2B1, we then examined the expression level of ERK, p38 and JNK after hnRNPA2B1 was knocked out. As shown in Figure 4A, there was no change in p38 and JNK signaling pathway, indicating a specific relationship with the ERK/MARK pathway. According to the previous studies, DUSPs can inactivate ERK1, 2 by dephosphorylating residues required for catalytic activity (Chappell et al., 2013; Ramkissoon et al., 2019; Martínez-Martínez et al., 2021). Then we selected the differential genes DUSP3/DUSP4/DUSP6 related to ERK dephosphorylation in the ERK/MAPK pathway and performed RT-qPCR to examine the expression of DUSP3/DUSP4/DUSP6 in control and hnRNPA2B1-KO cell lines. After hnRNPA2B1 was knocked out, the expression of these genes increased, resulting in a decrease in ERK phosphorylation (Figure 4B). Based on the DEGs results, there was no change in the ERK-related gene sets. It also explicated why only the p-ERK but not the total ERK protein level was affected after hnRNPA2B1 KO. Therefore, the regulation of ERK is at the post-translational level (Supplementary Figure 2B). To provide additional supporting evidence for this model, IHC staining of p-ERK, ERK and hnRNPA2B1 was performed in clinical colon cancer specimens. IHC staining results show that hnRNPA2B1 and p-ERK are partially co-expressed in colon cancer tissue due to tumor heterogeneity, which indicates that there is a regulatory basis between them (Supplementary Figure 2C). It also suggests that the regulatory relationship exists and targeting of hnRNPA2B1 is potential. Furthermore, application of small molecule inhibitors of the ERK pathway, U0126 and Trametinib, which inhibits MEK, immediately upstream of ERK, also significantly suppressed the expression of p-ERK as revealed by western blotting analyses. However, under this circumstance, expression of hnRNPA2B1 was unaffected (Figure 4C), suggesting that p-ERK does not regulate hnRNPA2B1 and that a positive feedback loop does not exist. In addition, we constructed the overexpression cell lines with HCT-8 and found that when hnRNPA2B1 is overexpressed, the expression of p-ERK was increased (Figure 4D). Meanwhile, the CCK8 assay also confirmed that the proliferation of HCT-8 increased after the hnRNPA2B1 overexpression, and the effects of hnRNPA2B1 were blocked after inhibiting the MAPK signaling (Figure 4E), which further supported that hnRNPA2B1 promotes colon cancer progression via the MAPK pathway. Taken together, these results support the model that hnRNPA2B1 positively regulates the activation of the ERK pathways to promote cell proliferation in colon cancer.

## Discussion

In this study, we demonstrate that hnRNPA2B1 promotes the progression of colon cancer by regulating the ERK/MAPK signaling pathway. First, by analyzing Oncomine and TCGA databases, we found that hnRNPA2B1 is highly expressed in colon cancer and is associated with poor disease-free interval and poor prognosis. Secondly, by western blotting and immunohistochemistry, upregulation of hnRNPA2B1 expression is confirmed in colon cancer cells and clinical samples. Thirdly, functional studies suggested that high expression of hnRNPA2B1 promotes cell proliferation and inhibits apoptosis *in vivo* and *in vitro*, which were further confirmed by RNA-seq analyses of hnRNPA2B1 knockout cell lines. Fourthly, we detected the expression of MAPK pathway related proteins by western blotting and found that the ERK/MAPK pathway gene expression is decreased after knockout hnRNPA2B1 in colon cancer SW480 cells, while the p38 and JNK signaling pathway gene expression is unchanged, suggesting a specific relationship between hnRNPA2B1 and the ERK/MAPK pathway. Moreover, IHC staining results show that hnRNPA2B1 and p-ERK are partially co-expressed in colon cancer tissue due to tumor heterogeneity, which indicates that there is a regulatory basis between them. It also suggests that the regulatory relationship is existing and targeting of hnRNPA2B1 is potential. By application of selective inhibitors of the ERK pathway, the expression of p-ERK is significantly downregulated, but hnRNPA2B1 expression is unaltered. It revealed that hnRNPA2B1 appeared to be an upstream regulator of the ERK/MAPK pathway. Finally, we constructed the overexpression cell lines in HCT-8 and found that the expression of p-ERK was increased and the proliferation of HCT-8 increased under the hnRNPA2B1 overexpression. It demonstrates that hnRNPA2B1-ERK/MAPK signal axis, which promotes colon cancer progression. Therefore, our data support the idea that hnRNPA2B1 acts as an upstream regulator of ERK and positively regulates its activity.

GO analyses showed that when the hnRNPA2B1 in high expression, multiple molecular functions related to post-translational modification were activated, such as “mRNA binding involved in posttranscriptional gene silencing,” “mRNA binding” and so on. And the results of GSEA also showed that cell proliferation is active. Besides, in the analyses of PPI protein interaction network, we found that gene networks related to alternative splicing in the control group, such as BPTF, KLHL22, HAUS7, etc., are significantly enriched. The PPI results and GO analyses results were identified that: hnRNPA2B1, as a member of the m6A reader, can participate in the process of RNA modification and alternative splicing. Those results were confirmed by the TCGA data too (Li et al., 2017). In tumors, alternative splicing helps to change and promote the pathophysiological characteristics of cancer (Ule and Blencowe, 2019). As one of the splicing factors, hnRNPA2B1 is upregulated in multiple tumors and affects their biological processes. Our sequencing results also show that hnRNPA2B1 is highly expressed the frequency of alternative splicing events in colon cancer is increased under the circumstances, which indicates alternative splicing may play an important role in colon cancer. The follow-up can start from the alternative splicing events to explore the pathogenesis of colon cancer.

However, there are a few important characteristics worth noting. On one hand, hnRNPA2B1 appears to be an important regulator of ERK/MAPK pathway, but whether or not it directly or indirectly activities the MAPK pathway gene in colon cancer remains to be determined. On the other hand, as an RNA-binding protein, hnRNPA2B1 plays an important role in post-transcriptional modification, including m6A modification, alternative splicing on mRNA (Ule and Blencowe, 2019; Wu et al., 2019; Hu et al., 2020), and so on. Currently, it is difficult to confirm what kinds of detailed mRNA modifications mediated by hnRNPA2B1 participate in colon cancer development and progress. In terms of m6A modification, sequencing such as MeRIP-seq and miCLIP is required for detailed analyses and discussion (Li et al., 2019). In terms of alternative splicing, our results in PPI and GO analyses both show that the alternative splicing under the condition of high expression of hnRNPA2B1 the regulation of excision is enhanced, and the process of tumor occurrence and development is also closely related to the post-transcriptional modification of alternative splicing, which means that the alternative splicing function in hnRNPA2B1 may play an important role in the progression of colon cancer, but it still need further experimental proof. Referring to immunity, hnRNPA2B1 can not only increase the antiviral innate immune response to DNA viruses, but also may be a potential marker for the early stage of the disease in the pathogenesis of rheumatoid arthritis, because immunity is essential for the occurrence and development of tumors. There is a question of does hnRNPA2B1 affects the immune status of colon cancer progression? All this requires us to conduct more in-depth research in subsequent experiments to answer the above questions and make an important contribution to determining new strategies for early diagnosis and prognosis of colon cancer. Answering the above questions in our subsequent experiments will make important contributions to identify novel strategies for early diagnosis and prognosis of colon cancer.

In conclusion, the present work is the first to show the expression, function and putative mechanism of hnRNPA2B1 with respect to cell proliferation and apoptosis in the setting of colon cancer and to propose that the ERK/MAPK pathway is activated by hnRNPA2B1 during this development process. hnRNPA2B1 is a potential diagnosis and therapeutic target for colon cancer, considering its role in post-transcriptional modification m6A modification and alternative splicing.

## Data Availability Statement

The publicly available datasets were analyzed in this study. High-throughput sequencing data were deposited at GEO under the accession number GEO: GSE169211.

## Ethics Statement

The animal study was reviewed and approved by the RenJi Hospital Institutional Animal Care and Ethics Committee (RJ2020-0601).

## Author Contributions

W-QG and HX conceived the concept and designed the study. JT and ZC performed most of the experiments, interpreted the data produced, and drafted the manuscript. QW gave advice for analyses. ZC and WH performed the statistical analyses. W-QG and HX edited the manuscript. All authors read and approved the final version of the manuscript.

## Conflict of Interest

The authors declare that the research was conducted in the absence of any commercial or financial relationships that could be construed as a potential conflict of interest.

## Publisher’s Note

All claims expressed in this article are solely those of the authors and do not necessarily represent those of their affiliated organizations, or those of the publisher, the editors and the reviewers. Any product that may be evaluated in this article, or claim that may be made by its manufacturer, is not guaranteed or endorsed by the publisher.

## Footnotes

1
https://portal.gdc.cancer.gov/

## References

[B1] Adhikari S. Xiao W. Zhao Y. L. Yang Y. G. (2016). m(6)A: signaling for mRNA splicing. *RNA Biol.* 13 756–759. 10.1080/15476286.2016.1201628 27351695 PMC5013988

[B2] Alarcon C. R. Goodarzi H. Lee H. Liu X. Tavazoie S. Tavazoie S. F. (2015). HNRNPA2B1 is a mediator of m(6)A-dependent nuclear RNA processing events. *Cell* 162 1299–1308. 10.1016/j.cell.2015.08.011 26321680 PMC4673968

[B3] Chappell J. Sun Y. Singh A. Dalton S. (2013). MYC/MAX control ERK signaling and pluripotency by regulation of dual-specificity phosphatases 2 and 7. *Genes Dev.* 27 725–733. 10.1101/gad.211300.112 23592794 PMC3639414

[B4] Chen C. Luo Y. He W. Zhao Y. Kong Y. Liu H. (2020). Exosomal long noncoding RNA LNMAT2 promotes lymphatic metastasis in bladder cancer. *J. Clin. Invest.* 130 404–421. 10.1172/JCI130892 31593555 PMC6934220

[B5] Chen W. Sun K. Zheng R. Zeng H. Zhang S. Xia C. (2018). Cancer incidence and mortality in China, 2014. *Chin. J. Cancer Res.* 30 1–12. 10.21147/j.issn.1000-9604.2018.01.01 29545714 PMC5842223

[B6] Chen W. Zheng R. Baade P. D. Zhang S. Zeng H. Bray F. (2016). Cancer statistics in China, 2015. *CA Cancer J. Clin.* 66 115–132. 10.3322/caac.21338 26808342

[B7] Fang J. Y. Richardson B. C. (2005). The MAPK signalling pathways and colorectal cancer. *Lancet Oncol.* 6 322–327. 10.1016/s1470-2045(05)70168-6 15863380

[B8] Geuens T. Bouhy D. Timmerman V. (2016). The hnRNP family: insights into their role in health and disease. *Hum. Genet.* 135 851–867. 10.1007/s00439-016-1683-5 27215579 PMC4947485

[B9] Gupta A. Yadav S. Pt A. Mishra J. Samaiya A. Panday R. K. (2020). The HNRNPA2B1-MST1R-Akt axis contributes to epithelial-to-mesenchymal transition in head and neck cancer. *Lab. Invest.* 100 1589–1601. 10.1038/s41374-020-0466-8 32669614

[B10] He L. Li J. Wang X. Ying Y. Xie H. Yan H. (2018). The dual role of N6-methyladenosine modification of RNAs is involved in human cancers. *J. Cell Mol. Med.* 22 4630–4639. 10.1111/jcmm.13804 30039919 PMC6156243

[B11] Hu X. Harvey S. E. Zheng R. Lyu J. Grzeskowiak C. L. Powell E. (2020). The RNA-binding protein AKAP8 suppresses tumor metastasis by antagonizing EMT-associated alternative splicing. *Nat. Commun.* 11:486. 10.1038/s41467-020-14304-1 31980632 PMC6981122

[B12] Kopetz S. Chang G. J. Overman M. J. Eng C. Sargent D. J. Larson D. W. (2009). Improved survival in metastatic colorectal cancer is associated with adoption of hepatic resection and improved chemotherapy. *J. Clin. Oncol.* 27 3677–3683. 10.1200/JCO.2008.20.5278 19470929 PMC2720081

[B13] Li T. Hu P. S. Zuo Z. Lin J. F. Li X. Wu Q. N. (2019). METTL3 facilitates tumor progression via an m(6)A-IGF2BP2-dependent mechanism in colorectal carcinoma. *Mol. Cancer* 18:112. 10.1186/s12943-019-1038-7 31230592 PMC6589893

[B14] Li Y. Sun N. Lu Z. Sun S. Huang J. Chen Z. (2017). Prognostic alternative mRNA splicing signature in non-small cell lung cancer. *Cancer Lett.* 393 40–51. 10.1016/j.canlet.2017.02.016 28223168

[B15] Liu F. Yang X. Geng M. Huang M. (2018). Targeting ERK, an Achilles’ Heel of the MAPK pathway, in cancer therapy. *Acta Pharm. Sin. B* 8 552–562. 10.1016/j.apsb.2018.01.008 30109180 PMC6089851

[B16] Liu Y. Li H. Liu F. Gao L. B. Han R. Chen C. (2020). Heterogeneous nuclear ribonucleoprotein A2/B1 is a negative regulator of human breast cancer metastasis by maintaining the balance of multiple genes and pathways. *EBioMedicine* 51:102583. 10.1016/j.ebiom.2019.11.044 31901866 PMC6948170

[B17] Martínez-Martínez D. Toledo Lobo M. V. Baquero P. Ropero S. Angulo J. C. Chiloeches A. (2021). Downregulation of Snail by DUSP1 impairs cell migration and invasion through the inactivation of JNK and ERK and is useful as a predictive factor in the prognosis of prostate cancer. *Cancers* 13:1158. 10.3390/cancers13051158 33800291 PMC7962644

[B18] Ramkissoon A. Chaney K. E. Milewski D. Williams K. B. Williams R. L. Choi K. (2019). Targeted inhibition of the dual specificity phosphatases DUSP1 and DUSP6 suppress mpnst growth via JNK. *Clin. Cancer Res.* 25 4117–4127. 10.1158/1078-0432.Ccr-18-3224 30936125 PMC6606396

[B19] Siegel R. L. Miller K. D. Jemal A. (2020). Cancer statistics, 2020. *CA Cancer J. Clin.* 70 7–30. 10.3322/caac.21590 31912902

[B20] Streitner C. Koster T. Simpson C. G. Shaw P. Danisman S. Brown J. W. (2012). An hnRNP-like RNA-binding protein affects alternative splicing by in vivo interaction with transcripts in Arabidopsis thaliana. *Nucleic Acids Res.* 40 11240–11255. 10.1093/nar/gks873 23042250 PMC3526319

[B21] Tang B. Liang W. Liao Y. Li Z. Wang Y. Yan C. (2019). PEA15 promotes liver metastasis of colorectal cancer by upregulating the ERK/MAPK signaling pathway. *Oncol. Rep.* 41 43–56. 10.3892/or.2018.6825 30365128 PMC6278416

[B22] Tauler J. Zudaire E. Liu H. Shih J. Mulshine J. L. (2010). hnRNP A2/B1 modulates epithelial-mesenchymal transition in lung cancer cell lines. *Cancer Res.* 70 7137–7147. 10.1158/0008-5472.CAN-10-0860 20807810

[B23] Ule J. Blencowe B. J. (2019). Alternative splicing regulatory networks: functions, mechanisms, and evolution. *Mol. Cell* 76 329–345. 10.1016/j.molcel.2019.09.017 31626751

[B24] Visvanathan A. Patil V. Arora A. Hegde A. S. Arivazhagan A. Santosh V. (2018). Essential role of METTL3-mediated m(6)A modification in glioma stem-like cells maintenance and radioresistance. *Oncogene* 37 522–533. 10.1038/onc.2017.351 28991227

[B25] Wang H. Liang L. Dong Q. Huan L. He J. Li B. (2018). Long noncoding RNA miR503HG, a prognostic indicator, inhibits tumor metastasis by regulating the HNRNPA2B1/NF-kappaB pathway in hepatocellular carcinoma. *Theranostics* 8 2814–2829. 10.7150/thno.23012 29774077 PMC5957011

[B26] Wang X. Lu Z. Gomez A. Hon G. C. Yue Y. Han D. (2014). N6-methyladenosine-dependent regulation of messenger RNA stability. *Nature* 505 117–120. 10.1038/nature12730 24284625 PMC3877715

[B27] Weng H. Huang H. Wu H. Qin X. Zhao B. S. Dong L. (2018). METTL14 inhibits hematopoietic stem/progenitor differentiation and promotes leukemogenesis via mRNA m(6)A modification. *Cell Stem Cell* 22 191–205e199. 10.1016/j.stem.2017.11.016 29290617 PMC5860916

[B28] Wu Y. Yang X. Chen Z. Tian L. Jiang G. Chen F. (2019). m(6)A-induced lncRNA RP11 triggers the dissemination of colorectal cancer cells via upregulation of Zeb1. *Mol. Cancer* 18:87. 10.1186/s12943-019-1014-2 30979372 PMC6461827

[B29] Yang Y. Wei Q. Tang Y. Yuanyuan W. Luo Q. Zhao H. (2020). Loss of hnRNPA2B1 inhibits malignant capability and promotes apoptosis via down-regulating Lin28B expression in ovarian cancer. *Cancer Lett.* 475 43–52. 10.1016/j.canlet.2020.01.029 32006618

[B30] Zhang B. Wu Q. Li B. Wang D. Wang L. Zhou Y. L. (2020). m(6)A regulator-mediated methylation modification patterns and tumor microenvironment infiltration characterization in gastric cancer. *Mol. Cancer* 19:53. 10.1186/s12943-020-01170-0 32164750 PMC7066851

[B31] Zhang C. Fu J. Zhou Y. (2019). A review in research progress concerning m6A methylation and immunoregulation. *Front. Immunol.* 10:922. 10.3389/fimmu.2019.00922 31080453 PMC6497756

[B32] Zhang H. Shi X. Huang T. Zhao X. Chen W. Gu N. (2020). Dynamic landscape and evolution of m6A methylation in human. *Nucleic Acids Res.* 48 6251–6264. 10.1093/nar/gkaa347 32406913 PMC7293016

