# Supplementary material for: hnRNPA2B1 Promotes Colon Cancer Progression via the MAPK Pathway
Source: Front Genet. 2021 Sep 22;12:666451. doi: 10.3389/fgene.2021.666451 (PMC8494201; doi:10.3389/fgene.2021.666451)
Supplement: Supplementary file 1 [file Data_Sheet_1.docx]

Supplementary Material

# Supplementary Figures


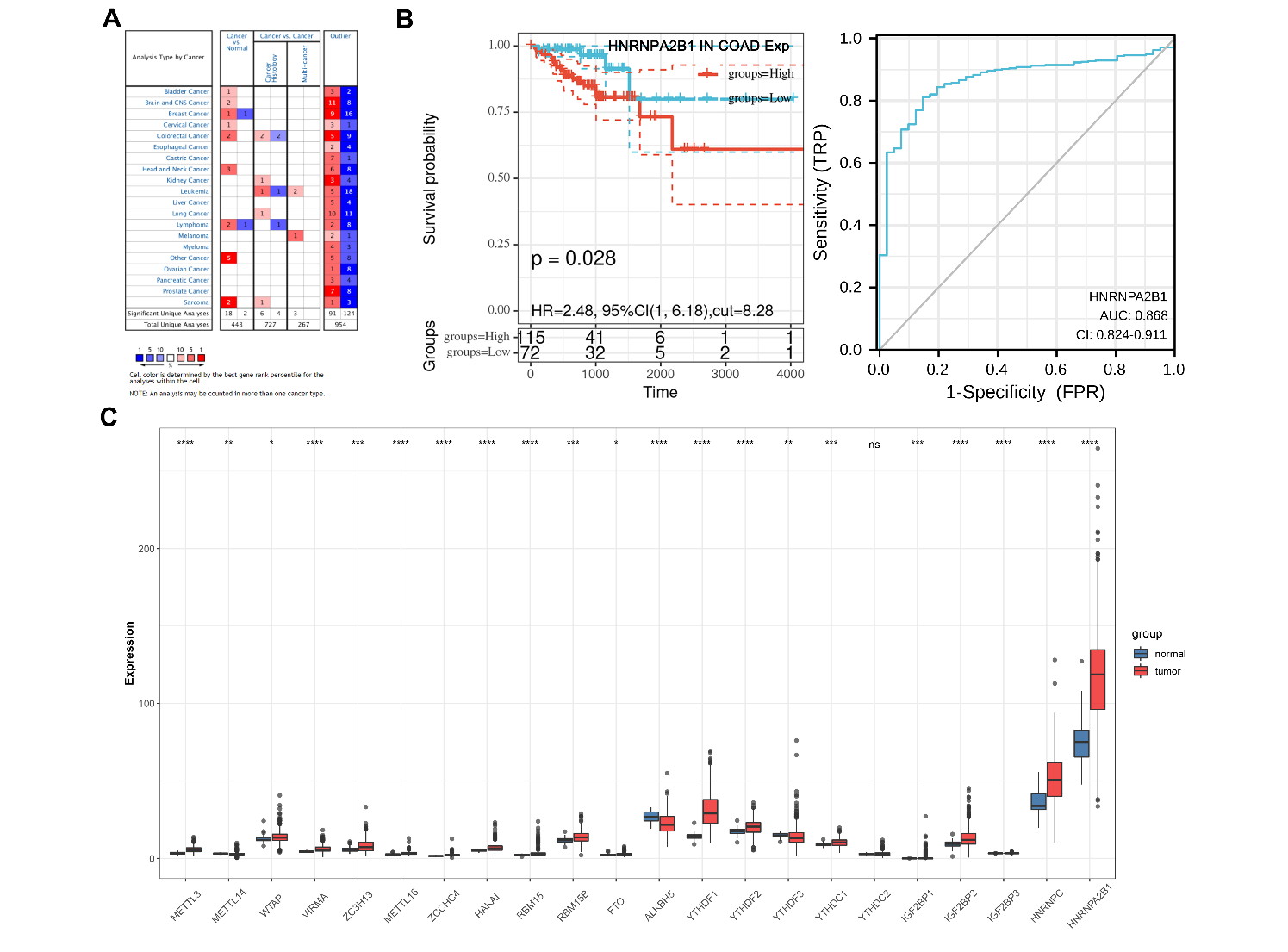


**Supplementary Figure 1.** **The expression of hnRNPA2B1 in colon cancer.** (A) Gene expression profiles in Oncomine database. The histology data are derived from Skrzypczak Colorectal 2 dataset. (B) The prognosis of hnRNPA2B1 in colon cancer (Disease-free interval). Kaplan-Meier survival curve (solid line) shows a 95% confidence interval (dashed line) (left). The x-axis represents the false positive rate (FPR), and the y-axis represents the true positive rate (TPR)(right). All data are from the TCGA data portal, including 480 colon cancer and 41 adjacent normal tissues. (C) Heatmap showing the m6A-related expression levels of 21 genes in colon cancer patient samples.

**
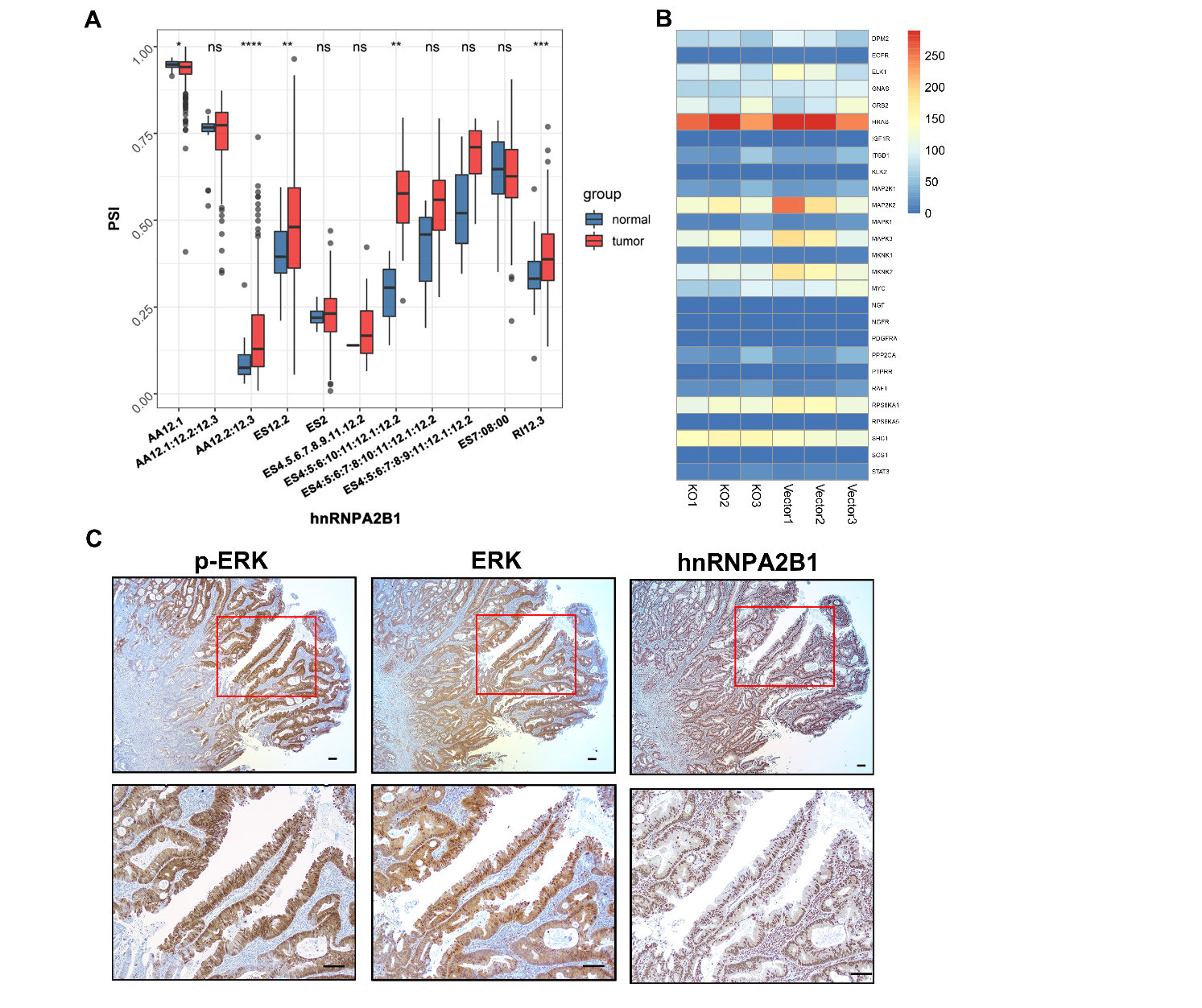
**

**Supplementary Figure 2.** (A) The effect of hnRNPA2B1 on alternative splicing in colon cancer. The data are derived from the TCGA database. AA: Alternate Acceptor Site. ES: Exon Skip. RI: Retained Intron. (B) The expression of ERK-related gene sets in hnRNPA2B1 knockout and control SW480 cells. (C) IHC staining of p-ERK, ERK, and hnRNPA2B1 was performed in clinical colon cancer tissues.
